# Prevalence, dimensionality and clinical relevance of self‐disturbances and psychotic‐like experiences in Polish young adults: a latent class analysis approach.

**DOI:** 10.1002/mpr.1809

**Published:** 2019-12-05

**Authors:** Renata Pionke, Piotr Gidzgier, Barnaby Nelson, Łukasz Gawęda

**Affiliations:** ^1^ Psychopathology and Early Intervention Lab, II Department of Psychiatry The Medical University of Warsaw Poland; ^2^ Center for Interdisciplinary Addiction Research, Department of Psychiatry and Psychotherapy University Medical Center Hamburg‐Eppendorf Hamburg Germany; ^3^ Orygen, The National Centre of Excellence in Youth Mental Health The University of Melbourne Parkville VIC Australia; ^4^ Centre for Youth Mental Health The University of Melbourne Parkville VIC Australia; ^5^ Clinical Neuroscience Lab Institute of Psychology, Polish Academy of Sciences Warsaw Poland

**Keywords:** latent class analysis, psychopathology, psychotic‐like experiences, self‐disturbances

## Abstract

**Objectives:**

We aimed to investigate latent classes of psychotic‐like experiences (PLEs) and self‐disturbances (SD) and to explore mutual overlapping between derived subgroups. Further, our goal was to investigate class membership relationship with an exposure to childhood trauma and different psychopathological factors such as cognitive biases, depression, insomnia, psychiatric diagnosis and lifetime suicidality.

**Methods:**

Participants consist of 3167 non‐clinical adults. We performed two latent class analyses (LCA), for PLEs and SD separately, to identify subgroups of individuals with different profiles on PLEs and SD. Associations between psychopathological factors and latent class membership were examined using multinomial logistic regression analysis.

**Results:**

LCA produced 5 classes within SD and 3 classes within PLEs. Class of the highest endorsement of SD showed 53% overlap with class of the highest endorsement of PLEs. The highest risk of belonging to High Class for both SD and PLEs was associated in particular with depression, cognitive biases and insomnia. Trauma emerged as a significant predictor only for PLEs classes.

**Conclusions:**

Our findings confirm that high PLEs and SD co‐occur and are concentrated in a relatively small number of individuals, at least in the general population. Their combination may capture the highest risk of psychosis in the general population.

## 1 INTRODUCTION

The growing interest and evidence for the hypothesis of the psychosis continuum causes more frequent search for risk factors among people from non‐clinical samples. One of the most recognized are psychotic‐like experiences (PLEs) and self‐disturbances (SD). Recent advances in the field have shown that both PLEs and SD among non‐clinical individuals are related to important risk factors including exposures to traumatic‐life events (Andorko et al., 2018; Gaweda et al., 2018a), cognitive biases (Gaweda et al., 2018b; Gaweda et al., 2018c; Moritz et al., 2017; Nelson, 2019), depression (Armando et al., 2010; Varghese et al., 2011), suicidal behaviors (Honings, Drukker, Groen, & van Os, 2016), insomnia (Cosgrave et al., 2018; Freeman et al., 2017; Koyanagi & Stickley, 2015) as well as to anomalies in dopamine synthesis (Howes, McCutcheon, Owen, & Murray, 2017). However, not much is known about the mutual relationship of PLEs and SD and how, taken together, they relate to known psychological risk factors for psychosis.

PLEs are phenomena that include subclinical illusions, hallucinations and thoughts on the continuum of delusions, for which insight is maintained, as well as phenomena associated with excessive expressiveness of the imagination (Yung et al., 2009). It is hypothesized that psychotic symptoms lie on the continuum, with PLEs on one end and psychotic disorders on the other (Yung et al., 2009). Therefore, there is a wider phenotype of psychosis in the general population and PLEs are one of its behavioral expressions (van Os, 2016). This provides the opportunity to study the mechanisms of psychosis amongst people with a high frequency of PLEs, but who are not afflicted by psychotic disorders.

SD are anomalous experiences of basic sense of self that have been considered as the core feature of schizophrenia spectrum disorders (e.g. Sass & Parnas, 2003) from the very beginning of modern psychiatry. SD refer to the so‐called minimal or basic self (‘ipseity'), which is thought to be the tacit, pre‐reflective level of selfhood and the ground or background of various aspects of conscious awareness (Sass & Parnas, 2003). It is an immediate (but implicit) conscious experience of oneself, a taken‐for‐granted ‘ownership' of experience that has an inbuilt first‐person perspective (‘for‐me‐ness'). There is a growing body of research indicating that SD are present not only in schizophrenia patients (Kean, 2009; Kean, 2010; Saks, 2007), but also in people who are at high clinical risk for psychosis (Davidsen, 2009; Nelson, Thompson, & Yung, 2012) and, in substantially attenuated form, in the non‐clinical population (Gaweda et al., 2019a; Gaweda et al., 2019b; Koren, Lacoua, Rothschild‐Yakar, & Parnas, 2016; Torbet, Schulze, Fiedler, & Reuter, 2015). They may also appear, but to a reduced degree, in other clinical populations such as panic disorder (Madeira et al., 2017) and depersonalisation disorder (Sass, Pienkos, Nelson, & Medford, 2013). Furthermore, it has been shown that SD precede the development of clinical symptoms of psychosis (Nelson et al., 2012).

Despite their great importance to the conceptualization of psychosis and psychosis risk, very little is known about the nature and latent patterns underlying these experiences. In particular, it is not clear what structures SD and PLEs have and to what extent they overlap with each other. Furthermore, it is not known what their specific relationships are with psychopathological risk factors. It has been shown that among people at high clinical risk for psychosis SD increase the risk of developing frank psychotic disorders (Nelson et al., 2012). Therefore, combining PLEs with SD as well as other psychopathological factors could improve the detection of people at risk of psychotic disorders in the general population (Gaweda et al., 2019a).

The multitude of screening questionnaires designed to investigate PLEs made it possible to study these experiences in large non‐clinical samples. Regarding SD, until recently, the lack of screening tools available hindered their reliable measurement. Thus, PLEs have been studied far more frequently than SD and are better understood. The gold standard in assessing SD is The Examination of Anomalous Self Experience (EASE) – a semi‐structured interview (Parnas et al., 2005) intended for examining schizophrenia spectrum disorders patients. It is a demanding tool and thus not suitable for a screening assessment in large populations. However, since 2017 there is a new tool available for measuring SD in a self‐report manner – The Inventory of Psychotic‐Like Anomalous Self‐Experiences (IPASE) (Cicero, Neis, Klaunig, & Trask, 2017). A very recent study has confirmed construct validity of the IPASE with correlation coefficients with the EASE reaching about 0.9 (Nelson et al., 2018), which suggests the IPASE may be a satisfactory proxy or initial assessment of SD. As a result, it is possible to study large non‐clinical populations and screen for the possible presence of SD as well as its significance and relationship with other psychopathological variables. Thereby, we can extend our knowledge about the mechanisms of SD by studying their presence and correlates in healthy people.

The first aim of the current study was to assess the prevalence of SD and PLEs in a non‐clinical sample of Polish young adults and to explore their dimensionality using latent class analysis (LCA). The second goal was to examine if and to what extent the identified classes of SD and PLEs overlap. SD and PLEs are related to broad psychopathology (Buckley, Miller, Lehrer, & Castle, 2009; Gaweda et al., 2018c; Honings et al., 2016). Therefore, the third goal was to investigate the relationship between LCA classes and important markers of risk for psychosis, namely an exposure to childhood trauma, cognitive biases, insomnia, suicidality, depression and psychiatric diagnosis.

## 2 METHODS

### 2.1 Participants

Three thousand one hundred and sixty‐seven people aged between 18 and 35 years (*M* = 26.67, *SD* = 4.78) were recruited from the non‐clinical population to participate in an online survey. Participants were enrolled from three large Polish cities: Warsaw (1 750 000 inhabitants), Krakow (770 000 inhabitants) and Wroclaw (640 000 inhabitants). A history of clinical diagnosis was screened with a self‐report questionnaire prepared for the study, and those who had a history of psychotic disorders or a history of addiction to any psychoactive substances were excluded. The study was approved by a local human research and ethics committee.

### 2.2 Measures

#### 2.2.1 Self‐disturbances (SD)

The Inventory of Psychotic‐Like Anomalous Self‐Experiences (IPASE) (Cicero et al., 2017) is a 57‐item self‐report questionnaire developed based on the phenomenological description of self‐disorders in schizophrenia spectrum disorders (Parnas et al., 2005). The items are clusterd into five dimensions, representing qualitatively different aspects of self‐disorder: 1) Cognition; 2) Self‐Awareness and Presence; 3) Consciousness; 4) Somatization and 5) Demarcation/Transitivism. Recently, the scale has been used in studies on psychosis proneness (Cicero et al., 2017) and among schizophrenia spectrum patients (Cicero, Klaunig, Trask, & Neis, 2016). We used a Polish version of the IPASE (Gaweda et al., 2018c). For this study, due to its nature (online screening) and the length of the scale we developed a short version of the IPASE that consists of 15 items. For every subscale we chose 3 items with top loadings. Cronbach's α for the total score calculated in our sample was 0.79.

#### 2.2.2 Psychotic‐like experiences (PLEs)

The sixteen‐item Prodromal Questionnaire (PQ‐16) (Ising et al., 2012) is a self‐report questionnaire to screen for psychosis risk operationalized as presence of PLEs. It is a shortened version of the 92‐item PQ and consists of items that assess perceptual abnormalities and hallucinations, unusual thought content, delusional ideas and paranoia as well as negative symptoms on a scale: present vs. non‐present – (true vs. false) which we modified to better reflect the frequency of PLEs. Specifically, we used a four‐point scale: ‘never', ‘sometimes', ‘often', and ‘almost always'. Most of the items in the PQ‐16 refer to attenuated positive psychotic symptoms. The PQ‐16 has satisfactory psychometric characteristics in assessment of PLEs with a specificity and sensitivity of 87% in discriminating patients meeting the criteria of UHR from those who do not meet UHR criteria (Ising et al., 2012). We used a Polish version of the questionnaire (Gaweda et al., 2018b). Cronbach's α for the total score calculated in our sample was 0.87.

#### 2.2.3 Cognitive biases

The Davos Assessment of Cognitive Biases Scale (DACOBS‐18) (van der Gaag et al., 2013) is a self‐report scale that assesses cognitive biases associated with psychosis. The Polish version of the questionnaire was prepared with a back‐translation procedure and has been used previously (Gaweda et al., 2018c; Gaweda, Prochwicz, & Cella, 2015). Due to the length of this scale and the nature of our study (online screening) we decided to use two subscales (Safety Behaviors and Attentional Biases) of the DACOBS‐18 that have been proven to be the best predictors of psychosis risk (Gaweda et al., 2018d). The two subscales included in the study consist of nine items. Cronbach's α for the total score was 0.81.

#### 2.2.4 Exposure to childhood trauma

To assess an exposure to trauma we focused on emotional, physical and sexual abuse that took place during childhood. Sexual trauma was measured with three items that we took from the Childhood Experience of Care and Abuse Questionnaire (CECA.Q) (Smith et al., 2002). It is a self‐report scale that measures the childhood experience of care and abuse. We also used two items from the Traumatic Experience Checklist (TEC) (Nijenhuis, Van der Hart, & Vanderlinden, 1999) to evaluate emotional neglect and abuse. The original version of the TEC is a 29‐item self‐report questionnaire addressing a variety of potentially traumatizing life events and has been used among patients with psychiatric disorders (Nijenhuis, Van der Hart, & Kruger, 2002). Additionally, we included one item to assess bullying and/or physical abuse from peers. Cronbach's α for the total score was 0.68.

#### 2.2.5 Depression

The Center for Epidemiologic Studies – Depression Scale (CESD‐R) (Eaton, Smith, Ybarra, Muntaner, & Tien, 2004) was used to measure current depressive symptomatology according to DSM‐IV‐R criteria for a major depressive episode and to identify possible cases of depressive disorders in the general population. Due to the length of this scale and the nature of our study we used a short version of the scale that consists of 5 items with the highest correlations with the total score and was validated in previous studies (Mętel et al., 2019, in press). We used a Polish version of the questionnaire (Jankowski, 2016). Cronbach's alpha calculated in our sample was 0.85.

#### 2.2.6 Insomnia

The Insomnia Severity Index (ISI) (Morin, 1993) was used to measure participants' perception of both nocturnal and diurnal symptoms of insomnia. It consists of seven items evaluating the perceived severity of difficulties initiating sleep, staying asleep, and early morning awakenings, satisfaction with current sleep pattern, interference with daily functioning, noticeability of impairment attributed to the sleep problem, and degree of distress caused by the sleep problem. Cronbach's α for the total score was 0.88.

#### 2.2.7 Lifetime suicidality

To evaluate suicidality, we used three questions designed to collect information on suicidal ideation (‘Have you ever seriously thought about committing suicide?'), plans (‘Have you ever made a plan for committing suicide?') and attempts (‘Have you ever attempted suicide?') that have been used in previous studies (Johnston, Pirkis, & Burgess, 2009). The sum of the answers (‘yes' or ‘no') to these three questions was used as a measure of lifetime suicidality. Cronbach's α for the total score was 0.74.

#### 2.2.8 Psychiatric diagnosis

A history of any psychiatric diagnosis was screened with a self‐report question (‘Have you ever been diagnosed with the psychiatric disorder?'). Demographic information (age, gender, education, professional situation) was also obtained.

### 2.3 Statistical analysis

LCA for PQ and IPASE separately was executed using Mplus 6.12 (Muthén & Muthén, 2012). In the case of the PQ‐16, we focused only on items targeting positive symptoms, therefore from sixteen items we excluded from the analysis two (1 and 7) that measure negative or depressive symptoms. Latent models between 2 and 6 classes characterized by a pattern of conditional probabilities were organized and compared. All models were estimated using default robust maximum likelihood estimator (Yuan & Bentler, 2000) with the assumption that all missing data were missing at random (Akaike, 1987). In order to confirm that the model reached the global maximum of likelihood and that the parameters were not estimated on local maxima solutions, the model was estimated using two different seeds and 500 random start values were utilized by 50 final stage optimizations.

We decided to choose models with the optimal number of classes after considering different model fit factors, including: the Akaike Information Criteria (AIC) (Akaike, 1987), the Bayesian Information Criteria (BIC) (Schwarz, 1978), the sample size adjusted Bayesian Information Criteria (ssaBIC) (Sclove, 1987) and the Lo–Mendell–Rubin's adjusted likelihood ratio test (LMRA‐LRT). Inferior values on the AIC, BIC and ssaBIC indicate better fit (Nylund, Asparouhov, & Muthén, 2007; Yang, 2006). As reported by Nylund et al. (2007) and Yang (2006), the most reliable indicator of fit is the BIC. It is confirmed that a model with a ten‐point lower BIC value has a 150:1 likelihood to be a more appropriate model and is recognized as a ‘very strong' evidence in comparison to a model with lower BIC value (Raftery, 1995). The LMRA‐LRT indicator determines if a latent model with one additional class is more suitable than a latent model with one less class. A non‐significant value (*p* > 0.05) for this test indicates that the latent model with one less class is the superior option. The bootstrapped likelihood ratio test (BLRT; (Nylund et al., 2007) was also taken into consideration. Its significant value (*p* < 0.05) indicates that the specified model is more suitable compared to a model with one class less. The entropy score was also taken into account as an indicator of classification quality of every particular model (Ramaswamy, DeSarbo, Reibstein, & Robinson, 1993). Entropy is described to be a standardized measure of how precisely participants are classified to a latent class. Superior classification is defined by values which approach 1 (Ramaswamy et al., 1993).

Due to the fact that we used the short version of the multidimensional IPASE scale, which has not yet been employed in a Polish sample, we decided to verify its factor structure using confirmatory factor analysis (CFA). After selecting the final number of latent classes for both IPASE and PQ scale, based on the probabilities for each item, we calculated the profile mean for each class and its corresponding 99% confidence interval. This allowed us to estimate more precisely which items stand out significantly from the profile mean and thus better capture the characteristics of the classes. When describing classes we also took into consideration the distribution of scores for each item. In addition, we analyzed the mutual overlap of PLEs and SD by comparing simultaneous occurrence of participants in PQ‐16 and IPASE latent classes.

In the next step, a series of multinomial univariate and multivariate logistic regressions were conducted to assess the association between class membership of the IPASE and PQ‐16 (posterior probabilities from the model were used to assign each participant to a class) and demographic factors (sex, age, years of education) as well as psychopathological variables (cognitive biases, depression, lifetime suicidality, insomnia, psychiatric diagnosis) and an exposure to childhood trauma. To analyze the percentage of explained variance in the outcome variables we used Nagelkerke pseudo R‐squared. With regard to continous psychopathological variables we divided participants into two groups using a cut‐off of 1.5 S.D. from the mean. The odds ratios indicate the expected increase/decrease in the likelihood of scoring positively on a given variable compared to the reference group, which is the class of the lowest symptom profile. First, we conducted univariate logistic regression analyses with each demographic and psychopathological variable. Then, we again performed analyses for psychopathological risk factors, but controlling for demographic variables as well as PLEs in analyses predicting class membership based on SD and, similarly, controlling for SD when we aimed to predict class membership based on PLEs. Using this method we were able to better investigate the independent contribution of each psychopathological variable taking into account their relationship with PLEs or SD. Finally, multivariate logistic regression analyses were carried out using all predictors included in univariate analyses in order to control for each variable.

## 3 RESULTS

### 3.1 Characteristics of the sample

Forty nine participants (1.52%, 14 with a diagnosis of schizophrenia and 35 with a history of addiction to psychoactive substances) were excluded from analyses. Table 1 presents sample characteristics.

**Table 1:** Sample characteristics

Gender	
Male	1143 (36.1%)
Female	2024 (63.9%)
Age	26.67 (4.78)
Education	
Primary	91 (2.9%)
Secondary	130 (4.1%)
Vocational	1055 (33.3%)
Incomplete higher	483 (15.3%)
Higher	1408 (44.5%)
Professional situation	
Study	1021 (32.2%)
Work	2390 (75.5%)
Unemployed	177 (5.6%)
Rent	18 (0.6%)
Psychiatric diagnosis	453 (14.3%)
Anxiety disorder	203 (6.4%)
Depression	318 (10%)
Bipolar disorder	14 (0.4%)
Obssessive‐compulsive disorder	37 (1.2%)
Personality disorder	61 (1.9%)
Eating disorder	68 (2.1%)
PQ‐16 (total score)	8.22 (6.11)
IPASE (total score)	7.29 (6.16)
DACOBS (total score)	26.08 (8.84)
ISI (total score)	7.48 (5.66)
CESD‐R (total score)	8.61 (3.56)
Childhood trauma (CT, total score)	1.58 (1.49)
Psychological distress	1640 (51.8%)
Lifetime suicidality	0.79 (1.02)
Ideations	1362 (43%)
Plans	850 (26.8%)
Attempts	294 (9.3%)

*Note:* PQ‐16, Prodromal Questionnaire; IPASE, Inventory of of Psychotic‐Like Anomalous Self‐Experiences; DACOBS, Davos Assessment of the Cognitive Biases Scale; ISI, Insomnia Severity Index; CESD‐R, Center for Epidemiologic Studies – Depression Scale

### 3.2 Confirmatory factor analysis of IPASE

CFA of the IPASE original structure revealed unsatisfactory model fit (RMSEA = 0.076, GFI = 0.93, CFI = 0.90, TLI = 0.88). Principal component analysis showed that item 9 from the Transitivism/Demarcation scale loads higher on the Self‐Awareness and Presence scale. After making this change, the model fit improved (RMSEA = 0.060, GFI = 0.96, CFI = 0.94, TLI = 0.93).

### 3.3 Latent class analysis of PQ‐16

The results of LCAs are presented in Table 2. The results of PQ‐16 subscale favored a model that consisted of 3 latent classes. The strongest indicator (BIC) suggested the three‐class solution and additionally, it was supported by LMRA‐LRT. The entropy value for the three‐class solution was 0.72, which is acceptable (Wang & Wang, 2012).

**Table 2:** Fit of the competing 2–6 class models for IPASE and PQ

IPASE	AIC	BIC	ssaBIC	Entropy	LMRA‐LRT (p)	BSLRT (p)
2c	22502.34	22690.22	22591.72	0.850	3686.851 (0.00)	3715.439 (0.000)
3c	21947.28	22232.13	22082.79	0.790	582.539 (0.00)	587.056 (0.000)
4c	21784.31	22166.13	21965.95	0.782	193.471 (0.039)	194.971 (0.000)
**5c**	**21686.29**	**22165.07**	**21914.05**	**0.804**	**129.024 (0.002)**	**130.025 (0.000)**
6c	21652.10	22227.85	21926.00	0.825	65.679 (0.408)	66.188 (0.000)
**PQ**						
2c	20650.37	20826.12	20733.98	0.811	2542.487 (0.000)	2563.515 (0.000)
**3c**	**20424.87**	**20691.53**	**20551.73**	**0.720**	**253.403 (0.0001)**	**255.499 (0.000)**
4c	20356.63	20714.20	20526.74	0.703	97.43 (0.132)	98.236 (0.000)
5c	20330.07	20778.55	20543.42	0.693	56.1 (0.077)	56.564 (0.000)
6c	20307.77	20847.16	20564.37	0.706	51.868 (0.457)	52.297 (0.000)

*Note:* AIC: Akaike Information Criterion; BIC: Bayesian Information Criterion; ssaBIC: sample size adjusted Bayesian Information Criterion; LMRA‐LRT: Lo–Mendell–Rubin adjusted likelihood ratio test; BSLRT (p): 2 times the Log‐likelihood difference and associated p‐value. Best model fits are bolded.

**Figure 1:**
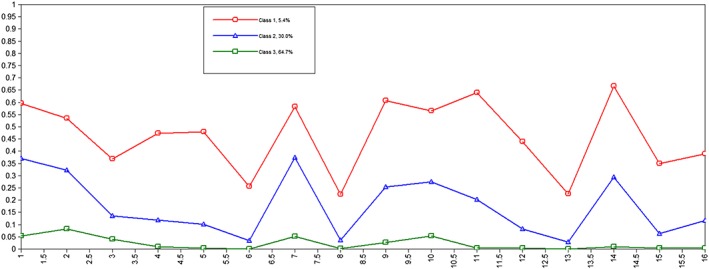
The latent profile plot for the three‐class PQ‐16 solution

Figure 1 presents the latent profile plot for the three‐class PQ‐16 solution. The first class was the largest, including 65.1% of the sample with no or very low endorsement of all PQ‐16 items. We termed it ‘Low Class'. This solution yielded also an intermediate class with 29.8% of all participants, which was named ‘Medium Class'. The third class was the smallest (5.2%). It was defined by the highest positive symptoms among all three classes, thus we named it ‘High Class'. Detailed data of class probabilities, profile means and 99% confidence intervals are presented in Supporting Information (Table S1).

### 3.4 Latent class analysis of IPASE

The results of LCA of IPASE items are shown in Table 2. All the indicators besides BSLRT were consistent in supporting the 5 class solution. The entropy value for this solution was 0.8 which indicated a high quality of class membership classification of participants in the study (Wang & Wang, 2012).

**Figure 2:**
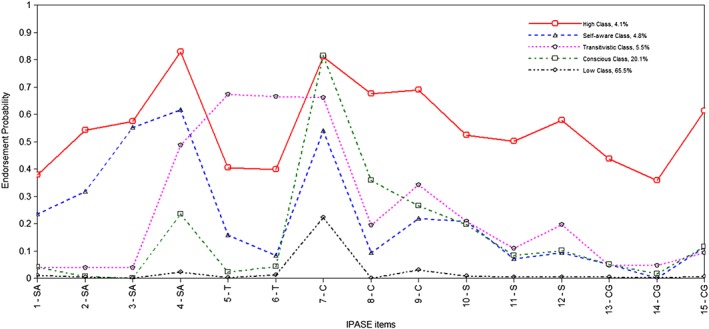
The latent profile plot for the five‐class IPASE solution

Figure 2 presents the latent profile plot for the five‐class IPASE solution. The largest class was ‘Low Class' with 65.5% of the sample and it was defined by very low scores on all items. For comparison, the class with the highest endorsement of all symptoms, which we named ‘High Class', included only 4.1% of participants. The solution yielded also three intermediate classess with different patterns of symptoms. We named one of them ‘Transitivistic Class' (5.5%) due to very high scores on two items that constitute Demarcation/Transitivism subscale of IPASE. These items not only leaned out from the profile mean of this class, but were also much higher than in ‘High Class'. As for classes other than ‘Transitivistic' and ‘High', scores on these two items were low. Next class was termed ‘Consciousness Class' (20.1%). It was characterised by low scores on all items except the ones from the Consciousness subscale. Two of them (items 7 and 8) significantly deviated from the profile mean and one (7) was as high as in High Class. The last one is ‘Self‐Aware Class' (4.8%). In this class occurrence of self‐awareness and presence symptoms were significantly higher than other symptoms or were higher than in other classes except High Class. Detailed data of class probabilities, profile means and 99% confidence intervals are presented as Supporting Information (Table S2).

### 3.5 Overlap of PQ‐16 and IPASE latent classes

In Supporting Information (Table S3) we present results of prevalence of participants in each latent class of PQ‐16 and IPASE. Of 125 participants belonging to High Class with the highest endorsement of SD, 66 are at the same time in High Class with the highest endorsement of PLEs. Thus, these high symptomatic classes overlap in nearly 53% cases. Also, in the High Class of SD, the percentage of participants belonging at the same time to Low Class of PQ‐16 is 8.8%. With regard to the classes of the lowest symptoms of both PLEs and SD the overlap is 85%. Furthermore, in the Low Class of SD, participants belonging at the same time to High Class of PLEs constitute 0.5% of this group.

**Table 3:** OR from univariate multinomial logistic regression analysis for psychopathological risk variables predicting IPASE LCA class membership, controlling for PLEs and demographic variables

	High vs. Low (ref.)		Consciousness vs. Low (ref.)		Transitivistic vs. Low (ref.)		Self‐Aware vs. Low (ref.)	
	OR (95% CI)	*p* value	OR (95% CI)	*p* value	OR (95% CI)	*p* value	OR (95% CI)	*p* value
PLEs	1.61 (1.54–1.68)	< 0.001	1.27 (1.24–1.30)	< 0.001	1.32 (1.27–1.36)	< 0.001	1.35 (1.30–1.40)	< 0.001

*Note:* OR, odds ratio; CI, 95% confidence interval.Adjusted for age, gender, years of education (not presented in the table) and PLEs.

### 3.6 Multinomial logistic regression analyses of IPASE

Univariate regression models with class membership of IPASE as the dependent variable showed that all predictors, except years of eductation, were statistically significant (see Table S4 in Supporting Information). After controlling for PLEs and demographic variables, odds ratio for all variables decreased and an exposure to trauma became insignificant predictor for all clusters. The data are presented in Table 3. The highest risk of belonging to High Class is associated with depression (OR = 7.63), cognitive biases (OR = 5.53) and insomnia (OR = 4.75).

**Table 4:** OR from univariate multinomial logistic regression analysis for psychopathological risk variables predicting PQ LCA class membership, controlling for SD and demographic variables

	High vs. Low (ref.)		Medium vs. Low (ref.)	
	OR (95% CI)	*p* value	OR (95% CI)	*p* value
SD	1.50 (1.45–1.56)	< 0.001	1.26 (1.23–1.28)	< 0.001
Cognitive biases	2.74 (1.63–4.62)	< 0.001	1.40 (0.98–2.00)	0.067
Exposure to trauma	2.27 (1.35–3.83)	0.002	1.59 (1.16–2.17)	0.003
Insomnia	3.25 (1.99–5.32)	< 0.001	1.83 (1.34–2.51)	< 0.001
Depression	2.96 (1.85–4.75)	< 0.001	2.27 (1.70–3.03)	< 0.001
Psychiatric diagnosis	2.35 (1.50–3.69)	< 0.001	1.66 (1.29–2.13)	< 0.001
Suicidality	2.03 (1.33–3.10)	0.001	1.61 (1.31–1.98)	< 0.001

*Note*: OR, odds ratio; CI, 95% confidence interval.Adjusted for age, gender, years of education (not presented in the table) and SD.

When taking into consideration as predictor only PLEs adjusted for demographic variables, they explain 40% variability in class membership of IPASE. Adding all other psychopathological variables and an exposure to trauma as predictors in multivariate regression causes increase in explained variance up to 45% (see Table S5 in Supporting Information).

### 3.7 Multinomial logistic regression analyses of PQ‐16

Univariate regression models with class membership of PQ‐16 as the dependent variable showed that all predictors, except gender and years of education, were statistically significant (see Table S6 in Supporting Information). After controlling for SD and demographic variables, all variables except cognitive biases in Medium Class remained significant. Detailed results are shown in Table 4. Similar to IPASE clusters, the highest risk of belonging to High Class was related to insomnia (OR = 3.25), depression (OR = 2.96) and cognitive biases (OR = 2.74).

When taking into consideration as predictor only SD adjusted for demographic variables, they explain 41% variability in class membership of PQ‐16. Adding all other psychopathological variables and an exposure to trauma as predictors in multivariate regression causes increase in explained variance up to 43.5% (see Table S7 in Supporting Information).

## 4 DISCUSSION

We applied a latent class analysis approach to identify groupings of individuals based on PLEs and SD in a non‐clinical sample of 3167 participants. LCA showed a three‐class solution of PQ‐16 items and a five‐class solution of IPASE items as models of the best fit. The subgroups of SD revealed an overlap with the subgroups of PLEs of nearly 53%. The remaining 47% represents the area of their independent, unshared variance. This finding, as well as the results of regression analyses, indicate that although SD and PLEs are closely related to each other and share common variance, they represent distinct constructs. Our findings are consistent with theoretical considerations and empirical studies on SD. According to Parnas and Hendriksen (2014) SD are considered to have a persisting, trait‐like nature with many schizophrenia spectrum disorders patients reporting that these experiences were already present during their childhood or early adolescence. SD not only precede the development of psychosis (Nelson et al., 2012; Parnas & Henriksen, 2014), but also persist when frank psychotic symptoms subside during remission (Nordgaard, Nilsson, Saebye, & Parnas, 2018; Parnas & Henriksen, 2014). Therefore, SD seem to represent a more fundamental psychopathology that gives rise to psychotic symptoms, which are fluctuating and less stable over time (Nelson, Yung, Bechdolf, & McGorry, 2008; Parnas & Handest, 2003; Sass, Borda, Madeira, Pienkos, & Nelson, 2018). The independence of the SD construct is also consistent with the proposal to include it as a diagnostic criterion for schizophrenia in the beta‐version of the ICD‐11 (Gaebel, Zielasek, & Falkai, 2015).

The three classes of PQ‐16 in our study were named: High, Medium and Low and they differ from each other mainly quantitatively. High Class was represented by the smallest number of participants (5.2%) and included the highest probability of endorsing all symptoms among the three classes. The items that were the highest in this class and deviate the most from the profile mean include a paranoid attitude, a sense of lack of control over one's own thoughts or ideas and excessive distinctness of thoughts. These subclinical symptoms seem to be the most pronounced in the group of individuals that represents the highest psychometric risk of developing psychosis. In contrast, Low Class was the largest (65.1%) with the probabilities close to zero. Our findings support the hypothesis that PLEs fall along a continuum (van Os, 2016; Yung et al., 2009). There have been several studies that investigated distribution of PLEs in the general population using LCA. Loch et al. (2017), using the original 92‐item PQ‐16, identified three quantitatively different classes, thus obtaining results very similar to the current findings. Shevlin, Murphy, Dorahy, and Adamson (2007) identified 4 different classes: psychosis class, a hallucinatory class, an intermediate class and a normative class. However, these four classes differed from each other only in item endorsement frequency, with no considerable qualitative difference, as in our case. Nonetheless, some other studies (Gale, Wells, McGee, & Browne, 2011; Ndetei et al., 2012) have shown other results indicating that, apart from classes of low and high endorsement of all PLEs, a hallucinatory class emerged as qualitatively different from others.

Results of a univariate regression analysis with PQ‐16 class membership as the dependent variable showed that all the psychopathological variables were sigificant predictors of belonging to High and Medium Class and were higher for High Class. However, when SD and demographic variables are being controlled in univariate regression, odds ratios for all variables decreased sharply (for example OR for depression declined from 18.21 to 2.96). Meanwhile, odds ratio for SD, however relatively small, remained at the same level. Multivariate analysis showed the same pattern of decresing odds ratio with psychiatric diagnosis and lifetime suicidality becoming insignificant predictors for High Class and cognitive biases and an exposure to trauma becoming insignificant for Medium Class. Furthermore, when considering only SD adjusted for demographic variables, they explain 41% of variability in class membership of PQ‐16. Adding all other psychopathological variables as predictors in multivariate regression causes increase in explained variance up to 43.5%. Considering the number of variables used as predictors, the increase in the explained variance is small. Taken together, the decrease of odds ratio for all psychopathological predictors after controlling for SD and the small change in the percentage of explaned variance we can conclude that SD are the most important risk marker for developing high level of PLEs among all other variables in our study.

To the best of our knowledge this is the first study to address the latent structure of SD in a non‐clinical sample using LCA. The data showed the presence of five distinct subgroups within the SD construct. Contrary to PQ‐16 LCA, classes differ from each other not only qunatitatively but also qualitatively suggesting that the underlying structure of SD is multifactorial. The analysis distinguished Low Class with the largest number of participants (65.5.%) and probabilities near zero as well as the smallest High Class (4.1%) with the highest endorsement of almost all symptoms. Three intermediate classes differed in severity of symptoms from subscales: Transitivism, Consciousness and Self‐Awareness, after which we named them. Results for the items from Cognition and Somatization Subscales were low in all classes, except for High Class. Therefore they may not have been useful in differantiating classes other than High Class.

The results of univariate regression analyses reveal a pattern according to which the highest odds ratio are observed in High Class and then decrease through Self‐Aware Class, Transitivistic Class and Consciousness Class. The exception are: psychiatric diagnosis, which highest odds ratio is associated with belonging to Self‐Aware Class, and suicidality with the third highest odds ratio in the Consciousness Class. After controlling for PLEs and demographic variables, the odds ratio for all variables decreased with an exposure to trauma becoming an insignificant predictor for all clusters and suicidality becoming insignificant for Transitivistic Class. In multivariate regression analyses odds ratio further decline with suicidality remaining significant only for Consciousness Class. As in the case with SD in predicting membership for PQ‐16 classes, odds ratio for PLEs, however relatively small, remained almost intact. Also, adding all psychopathological variables in multivariate analysis caused a relatively small increase in explained variance of 5%, which reveal high importance of PLEs for predicting IPASE classes.

When comparing the results of regression analyses for IPASE and PQ‐16 classes, the higher odds ratios of psychopathological factors are found for IPASE classes, which supports the notion of their independence. Constructs such as cognitive biases, depression, insomnia, suicidality and psychiatric diagnosis turned out to be related more strongly to SD than to PLEs. This is consistent with what schizophrenia patients report – experiences associated with SD are a source of greater distress than psychotic symptoms (Parnas & Henriksen, 2014), thus higher correlations with other psychopathological symptoms could be exptected. Regression analyses took into account PLEs or SD as a control variable, thus showing their individual contribution. With this control, an exposure to trauma remained a significant predictor for PQ‐16, but not for IPASE classes. This suggests that exposure to traumatic experiences is a specific (beyond variance shared with SD) predictor only for PLEs, which is in line with findings of the relationship between childhood trauma and PLEs (Kraan, Velthorst, Smit, de Haan, & van der Gaag, 2015; Varese et al., 2012). The strongest odds ratios for High Classes in case of both PLEs and SD turned out to be insomnia, cognitive biases and depression, which is in line with studies on psychosis and its risk states. The association between sleep difficulties and psychotic experiences is well established (Cosgrave et al., 2018). Study carried out by Freeman et al. (2017) showed that intervention targeting insomnia reduced paranoia and hallucinations which led to the conclusion that sleep problems may not only be a symptom but a causal factor in the occurrence of psychotic experiences. Cognitive biases are another well recognised cognitive factor related to the risk of psychosis both in clinical (Waters, Woodward, Allen, Aleman, & Sommer, 2012; Woodward, Moritz, Cuttler, & Whitman, 2006) and non‐clinical groups (Gaweda et al., 2018c; Moritz et al., 2017). It is postulated in Nelson and Sass (2017) recent theoretical model that cognitive biases such as source‐monitoring deficits could be neurocognitive correlates of SD. Preliminary data confirming their relationship in a group of ultra‐high risk of psychosis as well as among first‐episode schizophrenia patients has recently been published (Nelson et al., 2019). With regard to depression, a consistent body of research indicates not only that this diagnosis frequently co‐occurs with psychosis (Birchwood, Iqbal, Chadwick, & Trower, 2000; Siris et al., 2001), but also depressive symptoms are associated with psychotic experiences among ultra‐high risk individuals (Yung, Phillips, Yuen, & McGorry, 2004) and non‐clinical groups (Armando et al., 2010).

Our study has some limitations. First, the evaluation was based not on comprehensive psychiatric assessment, but on self‐report questionnaires. Therefore, items could be misunderstood by participants and, further, could contribute to over‐ or under‐rating of the experiences. Using self‐report question about having psychiatric diagnosis is also associated with the risk of self‐diagnosing without having a psychiatric disorder or not being aware of having one. Second, we used reduced versions of IPASE and PQ‐16 scales that have not yet been validated. We chose for the analyses only those items from PQ‐16 that describes positive PLEs. Thus, our results are limited as they do not apply to the entire spectrum of PLEs (e.g. negative symtpoms). Moreover, we focused only on the frequency scale of PQ‐16 and did not take into analyses the distress scale of this questionnaire. However, combining distress with frequency of PLEs in our opinion would hamper the clear interpretation of the results as well as their reference to the results of analyses carried out on IPASE scale, as it has no distress section. Finally, our study has a cross‐sectional design, which excludes causal inference. Longitudinal studies are required to verify whether psychopathological risk factors can predict further development of high levels of PLEs and SD as well as to test if the group of people with the highest endorsement of both PLEs and SD is also a group with the highest transition rate to psychosis in the future. Longitudinal design will also allow for investigation the dynamics of these variables.

To conclude, our results suggest that although SD and PLEs are related, they represent different constructs. Furthermore, our study indicated that classes with the highest profile of PLEs and SD were characterised by the highest severity of psychopatological factors associated with the psychosis risk. Indeed, research shown that high frequency of PLEs (Yung et al., 2003) and SD (Nelson et al., 2012) predict the onset of psychosis in ultra‐high risk groups. Thus, combining these risk markers into complex networks in which different factors dynamically interact with each factor (Isvoranu, Boyette, Guloksuz, & Borsboom, 2017) may have a potential to increase precision in detection individuals at the highest psychopathological risk of developing psychotic disorders (Nelson, McGorry, Wichers, Wigman, & Hartmann, 2017).

## CONFLICT OF INTEREST

The authors report no potential conflicts of interest.

## FINANCIAL SUPPORT

The study was supported by the OPUS grant from the National Science Center, Poland (2016/21/B/HS6/03210).

## Supporting information

Table S1. Probabilities of belonging to each PQ class
Table S2. Probabilities of belonging to each IPASE class
Table S3. Overlap between LCA of IPASE and PQ
Table S4. OR from univariate multinomial logistic regression analysis for demographic and psychopathological risk variables predicting IPASE LCA class membership
Table S5. OR from multivariate multinomial logistic regression analysis for psychopathological risk variables predicting IPASE LCA class membership
Table S6. OR from univariate multinomial logistic regression analysis for demographic and psychopathological risk variables predicting PQ LCA class membership
Table S7. OR from multivariate multinomial logistic regression analysis for psychopathological risk variables predicting PQ LCA class membership
