# Supplementary material for: Prevalence, dimensionality and clinical relevance of self‐disturbances and psychotic‐like experiences in Polish young adults: a latent class analysis approach
Source: Int J Methods Psychiatr Res. 2019 Dec 5;29(1):e1809. doi: 10.1002/mpr.1809 (PMC7051838; doi:10.1002/mpr.1809)
Supplement: Supplementary file 1 — Table S1. Probabilities of belonging to each PQ class Table S2. Probabilities of belonging to each IPASE class Table S3. Overlap between LCA of IPASE and PQ Table S4. OR from univariate multinomial logistic regression analysis for demographic and psychopathological risk variables predicting IPASE LCA class membership Table S5. OR from multivariate multinomial logistic regression analysis for psychopathological risk variables predicting IPASE LCA class membership Table S6. OR from univariate multinomial logistic regression analysis for demographic and psychopathological risk variables predicting PQ LCA class membership Table S7. OR from multivariate multinomial logistic regression analysis for psychopathological risk variables predicting PQ LCA class membership [file MPR-29-e1809-s001.docx]

**SUPPORTING INFORMATION**

Table 1. Probabilities of belonging to each PQ class

|  | Low  Class | Medium Class | High  Class |
| --- | --- | --- | --- |
| Profile Mean | 0.0165 | 0.1521 | 0.0165 |
| 99% CI | -0.0010, 0.0340 | 0.0699, 0.2343 | -0.001095, 0.034095 |
| PQ item | Probability | | |
| 2 | 0.077 | 0.339 | 0.534 |
| 3 | 0.036 | 0.147 | 0.373 |
| 4 | 0.008 | 0.12 | 0.499 |
| 5 | 0.002 | 0.108 | 0.487 |
| 6 | 0 | 0.034 | 0.268 |
| 8 | 0.002 | 0.04 | 0.23 |
| 9 | 0.021 | 0.274 | 0.596 |
| 10 | 0.046 | 0.297 | 0.556 |
| 11 | 0.004 | 0.213 | 0.621 |
| 12 | 0.006 | 0.076 | 0.457 |
| 13 | 0 | 0.029 | 0.241 |
| 14 | 0.024 | 0.265 | 0.676 |
| 15 | 0.001 | 0.068 | 0.361 |
| 16 | 0.004 | 0.12 | 0.388 |

Table 2. Probabilities of belonging to each IPASE class

|  | Low  Class | Consciousness  Class | Transitivistic Class | Self-Aware Class | High  Class |
| --- | --- | --- | --- | --- | --- |
| Profile Mean | 0,0221 | 0.1558 | 0.8506 | 0,2226 | 0,5538 |
| 99% CI | -0.0192, 0.0635 | -0.0009, 0.3125 | 0.0734, 0.4395 | 0.0757, 0.3694 | 0.4440, 0.6635 |
| IPASE item | Probability | | | | |
| 1 | 0.01 | 0.04 | 0.041 | 0.234 | 0.381 |
| 2 | 0.004 | 0.005 | 0.04 | 0.319 | 0.54 |
| 3 | 0 | 0 | 0.04 | 0.553 | 0.575 |
| 4 | 0.023 | 0.234 | 0.489 | 0.617 | 0.817 |
| 5 | 0.002 | 0.021 | 0.675 | 0.155 | 0.394 |
| 6 | 0.012 | 0.042 | 0.663 | 0.083 | 0.396 |
| 7 | 0.222 | 0.813 | 0.66 | 0.539 | 0.81 |
| 8 | 0.001 | 0.357 | 0.195 | 0.093 | 0.685 |
| 9 | 0.029 | 0.264 | 0.343 | 0.217 | 0.68 |
| 10 | 0.008 | 0.196 | 0.208 | 0.206 | 0.525 |
| 11 | 0.004 | 0.084 | 0.109 | 0.069 | 0.511 |
| 12 | 0.004 | 0.101 | 0.197 | 0.09 | 0.578 |
| 13 | 0.005 | 0.051 | 0.047 | 0.05 | 0.446 |
| 14 | 0.002 | 0.015 | 0.046 | 0 | 0.357 |
| 15 | 0.006 | 0.114 | 0.095 | 0.114 | 0.612 |

| LCA IPASE Class | LCA PQ Class | Frequency (%) |
| --- | --- | --- |
| Low | Low | 1871 (85.0) |
|  | Medium | 319 (14.5) |
|  | High | 11 (0.5) |
|  | *Overall* | 2201 (100.0) |
| Consciousness | Low | 256 (46.1) |
|  | Medium | 266 (47.9) |
|  | High | 33 (5.9) |
|  | *Overall* | 555 (100.0) |
| Transitivistic | Low | 71 (41.0) |
|  | Medium | 84 (48.6) |
|  | High | 18 (10.4) |
|  | *Overall* | 173 (100.0) |
| Self-Aware | Low | 39 (34.5) |
|  | Medium | 58 (51.3) |
|  | High | 16 (14.2) |
|  | *Overall* | 113 (100.0) |
| High | Low | 11 (8.8) |
|  | Medium | 48 (38.4) |
|  | High | 66 (52.8) |
|  | *Overall* | 125 (100.0) |

Table 3. Overlap between LCA of IPASE and PQ

Table 4. OR from univariate multinomial logistic regression analysis for demographic and psychopathological risk variables predicting IPASE LCA class membership

|  | High vs. Low (ref.) | | Consciousness vs. Low (ref.) | | Transitivistic vs. Low (ref.) | | Self-Aware vs. Low (ref.) | |
| --- | --- | --- | --- | --- | --- | --- | --- | --- |
|  | OR (95% CI) | *p* value | OR (95% CI) | *p* value | OR (95% CI) | *p* value | OR (95% CI) | *p* value |
| Sex (female) | 1.54 (1.04 - 2.29) | 0.033 | 1.23 (1.01 – 1.49) | 0.041 | 1.72 (1.21 – 2.44) | 0.002 | 1.80 (1.17 - 2.78) | 0.007 |
| Age | 0.95 (0.92 – 0.99) | 0.010 | 0.96 (0.94 – 0.98) | < 0.001 | 0.93 (0.90 – 0.97) | < 0.001 | 0.94 (0.90 – 0.98) | 0.002 |
| Years of education | 0.97 (0.90 – 1.04) | 0.358 | 1.01 (0.97 – 1.04) | 0.745 | 0.99 (0.94 – 1.06) | 0.917 | 0.99 (0.92 – 1.06) | 0.779 |
| PLEs | 1.61 (1.54 - 1.68) | < 0.001 | 1.27 (1.24 – 1.30) | < 0.001 | 1.32 (1.28 – 1.36) | < 0.001 | 1.35 (1.30 – 1.40) | < 0.001 |
| Cognitive biases | 20.47 (13.39 – 31.30) | <0.001 | 3.48 (2.44 – 4.96) | < 0.001 | 5.32 (3.32 – 8.52) | < 0.001 | 9.47 (5.82 – 15.39) | < 0.001 |
| Exposure to trauma | 5.67 (3.65 – 8.80) | < 0.001 | 2.42 (1.78 – 3.28) | < 0.001 | 2.65 (1.66 – 4.23) | < 0.001 | 4.21 (2.57 - 6.88) | < 0.001 |
| Insomnia | 17.47 (11.56 – 26.41) | < 0.001 | 4.78 (3.51 – 6.50) | < 0.001 | 5.39 (3.47 – 8.35) | < 0.001 | 6.74 (4.12 – 11.03) | < 0.001 |
| Depression | 32.04 (21.11 – 48.65) | < 0.001 | 7.57 (5.60 – 10.23) | < 0.001 | 13.90 (9.45 – 20.46) | < 0.001 | 16.50 (10.61 - 25.65) | < 0.001 |
| Psychiatric diagnosis | 6.83 (4.62 – 10.10) | < 0.001 | 3.19 (2.48 – 4.09) | < 0.001 | 4.09 (2.83 – 5.90) | < 0.001 | 8.39 (5.62 – 12.54) | < 0.001 |
| Suicidality | 9.08 (6.18 – 13.35) | < 0.001 | 3.25 (2.65 – 3.97) | < 0.001 | 2.94 (2.12 – 4.07) | < 0.001 | 5.20 (3.54 – 7.65) | < 0.001 |

*Note:* OR, odds ratio; CI, 95% confidence interval.

Table 5. OR from multivariate multinomial logistic regression analysis for psychopathological risk variables predicting IPASE LCA class membership

|  | High vs. Low (ref.) | | Consciousness vs. Low (ref.) | | Transitivistic vs. Low (ref.) | | Self-Aware vs. Low (ref.) | |
| --- | --- | --- | --- | --- | --- | --- | --- | --- |
|  | OR (95% CI) | *p* value | OR (95% CI) | *p* value | OR (95% CI) | *p* value | OR (95% CI) | *p* value |
| PLEs | 1.50 (1.44 – 1.57) | < 0.001 | 1.23 (1.20 – 1.26) | < 0.001 | 1.27 (1.23 – 1.31) | < 0.001 | 1.27 (1.22 – 1.32) | < 0.001 |
| Cognitive biases | 3.75 (2.07 – 6.80) | < 0.001 | 1.60 (1.04 – 2.47) | 0.034 | 2.06 (1.18 – 3.61) | 0.011 | 3.20 (1.78 – 5.74) | < 0.001 |
| Exposure to trauma | 1.08 (0.60 – 1.98) | 0.789 | 0.91 (0.64 – 1.31) | 0.630 | 0.88 (0.52 – 1.49) | 0.635 | 1.18 (0.67 – 2.08) | 0.564 |
| Insomnia | 3.13 (1.80 – 5.46) | < 0.001 | 2.02 (1.42 – 2.89) | < 0.001 | 1.84 (1.11 – 3.03) | 0.017 | 1.86 (1.06 – 3.26) | 0.031 |
| Depression | 5.24 (3.07 – 8.94) | < 0.001 | 2.84 (2.02 – 3.98) | < 0.001 | 4.85 (3.15 – 7.47) | < 0.001 | 4.45 (2.71 – 7.31) | < 0.001 |
| Psychiatric diagnosis | 1.80 (1.06 – 3.05) | 0.030 | 1.48 (1.10 – 1.99) | 0.009 | 1.77 (1.16 – 2.72) | 0.008 | 3.17 (1.99 – 5.05) | < 0.001 |
| Suicidality | 1.65 (0.99 – 2.74) | 0.054 | 1.31 (1.02 – 1.67) | 0.031 | 0.88 (0.60 – 1.30) | 0.536 | 1.22 (0.78 – 1.93) | 0.383 |

*Note*: OR, odds ratio; CI, 95% confidence interval.
Adjusted for age, gender and years of education (not presented in the table).

Table 6. OR from univariate multinomial logistic regression analysis for demographic and psychopathological risk variables predicting PQ LCA class membership

|  | High vs. Low (ref.) | | Medium vs. Low (ref.) | |
| --- | --- | --- | --- | --- |
|  | OR (95% CI) | *p* value | OR (95% CI) | *p* value |
| Sex (female) | 1.30 (0.91 – 1.87) | 0.150 | 1.17 (0.98 – 1.39) | 0.076 |
| Age | 0.95 (0.92 – 0.99) | 0.009 | 0.94 (0.92 – 0.95) | < 0.001 |
| Years of education | 0.97 (0.91 – 1.03) | 0.346 | 0.98 (0.95 – 1.01) | 0.181 |
| SD | 1.51 (1.45 – 1.56) | < 0.001 | 1.26 (1.24 – 1.29) | < 0.001 |
| Cognitive biases | 15.35 (10.30 – 22.87) | <0.001 | 3.64 (2.69 – 4.91) | < 0.001 |
| Exposure to trauma | 6.25 (4.15 – 9.42) | < 0.001 | 2.81 (2.15 – 3.69) | < 0.001 |
| Insomnia | 13.36 (9.09 – 19.64) | < 0.001 | 3.85 (2.93 – 5.05) | < 0.001 |
| Depression | 18.21 (12.47 – 26.59) | < 0.001 | 6.25 (4.86 – 8.04) | < 0.001 |
| Psychiatric diagnosis | 5.90 (4.10 – 8.50) | < 0.001 | 3.00 (2.41 – 3.72) | < 0.001 |
| Suicidality | 6.60 (4.65 – 9.35) | < 0.001 | 3.32 (2.78 – 3.97) | < 0.001 |

*Note:* OR, odds ratio; CI, 95% confidence interval.

Table 7. OR from multivariate multinomial logistic regression analysis for psychopathological risk variables predicting PQ LCA class membership

|  | High vs. Low (ref.) | | Medium vs. Low (ref.) | |
| --- | --- | --- | --- | --- |
|  | OR (95% CI) | *p* value | OR (95% CI) | *p* value |
| SD | 1.42 (1.37 – 1.48) | < 0.001 | 1.22 (1.19 – 1.24) | < 0.001 |
| Cognitive biases | 2.06 (1.20 – 3.54) | 0.009 | 1.17 (0.81 – 1.70) | 0.406 |
| Exposure to trauma | 1.88 (1.10 – 3.22) | 0.021 | 1.37 (0.99 – 1.89) | 0.055 |
| Insomnia | 2.42 (1.46 – 4.03) | 0.001 | 1.55 (1.12 – 2.14) | 0.008 |
| Depression | 2.14 (1.31 – 3.50) | 0.002 | 1.93 (1.44 – 2.60) | < 0.001 |
| Psychiatric diagnosis | 1.61 (0.99 – 2.62) | 0.055 | 1.31 (1.01 – 1.71) | 0.045 |
| Suicidality | 1.36 (0.86 – 2.16) | 0.189 | 1.35 (1.08 – 1.68) | 0.008 |

*Note:* OR, odds ratio; CI, 95% confidence interval.
Adjusted for age, gender and years of education (not presented in the table).
